# The impact of microRNA-122 and its target gene Sestrin-2 on the protective effect of ghrelin in angiotensin II-induced cardiomyocyte apoptosis†

**DOI:** 10.1039/c7ra13028g

**Published:** 2018-03-13

**Authors:** Xiaotong Wang, Chunyan Yang, Xueyan Liu, Ping Yang

**Affiliations:** The Department of Cardiology, China-Japan Union Hospital, Jilin University Changchun 130011 Jilin China pyang@jlu.edu.cn

## Abstract

Ghrelin with *n*-octanoylated serine 3 residue is a peptide hormone with well-known cardioprotective properties. MicroRNA-122 is associated with the pathogenesis of many cardiovascular diseases, including apoptosis and was found highly increased in our previous rat model of post-myocardial infarction heart failure. In this study, we aimed to identify the target gene of microRNA-122 and to evaluate their impacts on the protective effect of acylated ghrelin in angiotensin II-induced apoptosis. The results showed that microRNA-122 was upregulated in the angiotensin II administration group accompanied by increased cell apoptosis, which were both reversed by ghrelin. Furthermore, microRNA-122 mimics upregulated numerous pro-apoptotic genes and increased apoptosis. The luciferase activity assay revealed Sestrin-2 as a direct target of microRNA-122. The expression of Sestrin-2 was downregulated by angiotensin II and upregulated by co-treatment with ghrelin. Inhibition of microRNA-122 and overexpression of Sestrin-2 alleviated apoptosis which was further reduced upon administered of ghrelin. Together, these results indicated that Sestrin-2 expression is inhibited by microRNA-122 and that this inhibition is involved in the protective effect of ghrelin and angiotensin II-induced apoptosis. We also found that microRNA-122 influenced several apoptosis pathways including the caspase cascade reaction and death receptor-mediated pathways. Collectively, our data reveal that microRNA-122 and its target gene Sestrin-2, under the regulation of angiotensin II and ghrelin, are important players in cardiomyocyte apoptosis. We therefore believe that microRNA-122 and Sestrin-2 can be developed as potential therapeutic targets against apoptosis in cardiovascular diseases.

## Abstract 2

Inhibition of microRNA-122 and overexpression of Sestrin-2 alleviated angiotensin II-induced cardiomyocyte apoptosis and enhanced the protective effect of ghrelin.

## Graphical abstract

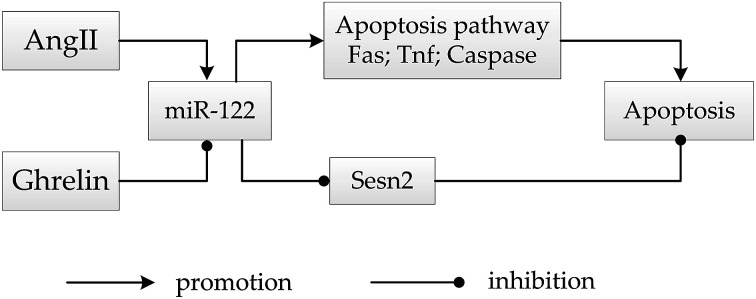

## Introduction

Myocardial apoptosis plays a critical role in acute myocardial infarction and many other cardiovascular diseases associated with heart failure. Loss of cardiac myocytes, which are terminal differentiated cells, is lethal and results in contractile tissue loss, reparative fibrosis, and subsequent left ventricular remodeling, all of which contribute to left ventricular dysfunction.^1–3^ Such heart dysfunction in turn leads to more cardiomyocyte apoptosis.^4^ Therefore, the development of methods for ameliorating cardiac myocyte apoptosis has drawn the interest of many researchers worldwide.

Angiotensin II (Ang II), a peptide derived from angiotensin I, is the most important component of the renin-angiotensin system (RAS). It has vasoconstriction properties and has been found to be associated with cell apoptosis, hypertrophy, and cardiac remodeling.^5^ Ghrelin, a peptide hormone isolated from the stomach, is considered to regulate growth hormone (GH) secretion as the endogenous ligand for growth hormone secretagogue receptor (GHS-R), in which process *n*-octanoylated serine 3 residue of ghrelin is necessary.^6^ Besides its functions in regulating energy homeostasis, ghrelin and its receptor possibly have a function in the heart as well,^7^ since they were found to be expressed in the heart tissue, such as in cardiac ventricles and blood vessels.^8^ Ghrelin has been reported to confer cardioprotective effects such as ameliorating left ventricular dysfunction and remodeling, protecting the heart against ischemia/reperfusion injury, improving energy metabolism in heart failure, and inhibiting cardiomyocyte apoptosis through the ERK1/2 and PI3-kinase/AKT signaling pathways.^9^ These cardioprotective effects make ghrelin a novel pharmacological target for the treatment of cardiovascular diseases. However, the molecular mechanisms underlying these effects of ghrelin are still unknown. Recent studies have shown that ghrelin can upregulate microRNAs (miRNAs) and can in turn be regulated by miRNAs.^10,11^ However, the target miRNAs of ghrelin in inhibiting Ang II-induced apoptosis and the mechanisms underlying their role in cardiovascular diseases, especially in cardiomyocytes apoptosis, have not been fully elucidated.

MicroRNAs (miRNAs) belong to a class of small non-coding RNAs (about 20–25 nucleotides) that negatively regulate the translation of target genes and/or promote the degradation of the transcribed mRNAs through binding to the 3′ untranslated region (UTR) of the miRNA targets.^12,13^ MiRNAs play crucial roles in the regulation of gene expression: approximately 1/3rd of known human genes are regulated by miRNAs.^14^ In particular, in cardiovascular diseases, miRNAs critically involve in regulating cellular processes including apoptosis, proliferation, fibrosis, and many other pathological pathways.^15–19^ Moreover, the clinical application of miRNAs as diagnostic and prognostic targets has attracted a lot of research attention.^18^

MicroRNA-122 (miR-122) is conserved in vertebrates, and although it is highly expressed in the liver, it has recently been found to be associated with different pathogenesis in cardiovascular diseases, including apoptosis and fibrosis.^20–23^ In our previous study,^24^ the differential miRNA profile in a rat model of post-infarction heart failure showed that miR-122, as one of the most abundantly expressed miRNAs, is markedly upregulated during the development of heart failure, which revealed its potential role in this complex pathological process. Furthermore, miR-122 has been applied as a novel biomarker for many cardiovascular diseases such as acute coronary syndrome^25–28^ and as a predictor of outcome.^29^ However, whether miR-122 involves in Ang II-induced apoptosis and the mechanisms by which miR-122 contributes to apoptosis remain unclear.

Sestrin-2 (Sesn2), an important member of the sestrin family, can be activated by the transcriptional factor p53. The encoded proteins are called stress-inducible proteins, since they can protect cells against various insults such as oxidative stress and DNA damage.^30^ In cardiovascular diseases, Sesn2 exerts cytoprotective effects in energetic stress-induced cell death, Ang II-induced endothelial toxicity, and myocardial ischemia,^31–33^ all of which can result in heart failure.

H9c2 cells derived from the embryonic rat heart are ideal for signal transduction research due to their division property and have been used as a cell model in several studies on apoptosis.^34,35^ In this study, we aimed to identify the target gene of miR-122 and to evaluate their impacts on the protective effect of ghrelin in Ang II-induced apoptosis. We found that miR-122 expression was upregulated in H9c2 cells and this upregulation was accompanied by increased cell apoptosis induced by Ang II treatment; however, both these effects could be ameliorated by ghrelin pretreatment. PCR array was performed to further explore the pathways influenced by miR-122 in apoptosis, and the target gene of miR-122 was identified by luciferase reporter assay. Additionally, miR-122 mimics and inhibitors, as well as a Sesn2 overexpression vector (pEX-Sesn2), were transfected into H9c2 cells to investigate their impacts on the cardioprotective effect of ghrelin on Ang II-induced apoptosis. The study reveals miR-122 and its target gene that play important roles in cardiomyocyte apoptosis and can therefore be used as a therapeutic target.

## Experimental

### Materials

H9c2, a rat cardiac myoblast cell line, was obtained from American Type Culture Collection (ATCC, Cat# CRL-1446, RRID: CVCL_0286). Dulbecco Minimal Essential Medium (DMEM) and fetal bovine serum (FBS) were purchased from Gibco (Cat# 11965-092, 10099-141). 3-(4,5-Dimethyl-2-thiazolyl)-2,5-diphenyl-2*H*-tetrazolium bromide (MTT) was obtained from Sigma-Aldrich (Cat# M2128). Ang II was purchased from Tocris Bioscience (Cat# 1158). Ghrelin was obtained from Phoenix Biotech (Cat# 031-31). The Annexin V-fluorescein isothiocyanate (FITC) apoptosis detection kit and BCA Protein Quantitation Assay were purchased from KeyGen Biotech (Cat# KGA108, KGP902). PrimeScript™ RT reagent kit and SYBR fluorescent quantitation kit were provided by Takara (Cat# RR047A, RR420A). The Rat Apoptosis RT^2^ Profiler™ PCR Array and other related reagents were obtained from QIAGEN Bioinformatics (Cat# PARN-012Z). The Fugene transfection reagent and Dual-Luciferase Reporter Assay System were purchased from Promega (Cat# E2311, E1910). Primary antibodies against SESN2 (Cat# ab178518), β-ACTIN (Cat# ab16039), and horseradish peroxidase (HRP)-conjugated goat anti-rabbit IgG secondary antibody (Cat# ab6721) were purchased from Abcam and the details of these antibodies are described in Table 1.

**Table 1:** The information of antibodies used in western blot

Target	Name of antibody	Manufacturer, catalog no.	Species raised in mono/polyclonal	Dilution used
SESN2	Anti-SESN2 antibody	Abcam, Cat# ab178518	Rabbit monoclonal antibody	1 : 1000
β-ACTIN	Anti-BETA ACTIN antibody	Abcam, Cat# ab16039	Mouse monoclonal antibody	1 : 1000

### Cell culture, transfection, and treatments

H9c2 cells were cultured in DMEM supplemented with 10% FBS, 100 U mL^−1^ penicillin, and 100 mg mL^−1^ streptomycin in a 5% CO_2_/95% air humidified incubator at 37 °C. The cells were then seeded in 6-well plates at a density of 1 × 10^5^ per well during the logarithmic growth phase. When the cells reached 80% confluence, the miRNA negative control (NC), mimics, and inhibitors (20 μM) synthesized by Gene Pharma were transfected into the cells using Fugene HD reagents. Sesn2 gain-of-function experiments were conducted using the overexpression vector pEX-Sesn2 (Sesn2 cDNA was inserted into the pEX-4 vector provided by Gene Pharma). After the transfection was complete (usually after 12–24 h), the culture medium (control) and ghrelin (100 nM), Ang II (100 nM), or both were added to the culture dishes, followed by incubation for 24 h. Total RNA and proteins were extracted for qPCR and western blot analysis, respectively.

### Measurement of cell viability using the MTT assay

The MTT assay works based on the transformation of exogenous MTT into an insoluble formazan crystal by mitochondrial dehydrogenases of living cells. In this study, the H9c2 cells were cultured at a density of 5 × 10^3^ cells per well into 96-well plates. After 24 h, the cells were incubated with culture medium or treated with Ang II (100 nM) for different durations (from 0 h to 48 h) with or without ghrelin. Next, MTT (5 mg mL^−1^) was added to the cells (20 μL per well) for 4 h. Finally, the culture medium was removed from the wells, and dimethyl sulfoxide was added into the wells to resolve the formazan crystals (150 μL per well). After agitation at room temperature for 10 min, the absorbance at 490 nm of each well was measured using a spectrophotometer microplate reader. The viability was defined as the percentage of absorbance of the treated cells *versus* that of the untreated cells.

### Assessment of apoptosis by flow cytometry using annexin V-FITC/PI staining

To measure apoptosis, the H9c2 cells were harvested using TrypleE (Gibco, Cat# 12604021) and washed with PBS 3 times. Then, 500 μL binding buffer mixed with 5 μL annexin V-FITC and propidium iodide (PI) was added per 1 × 10^5^ cells. The percentage of apoptotic cells was then assessed by flow cytometry.

### RNA extraction and real-time quantitative PCR

Total RNA was extracted using the RNeasy® mini kit (QIAGEN). cDNA was synthesized using the RT reagent kit, and real-time quantitative PCR was performed with the Stratagene Mx3005p Real-Time PCR System (Agilent, CA, USA) using SYBR Green Master Mix. The PCR program was as follows: 1 cycle of 95 °C for 10 min; 40 cycles of 95 °C for 30 s, and 60 °C for 1 min; and 1 cycle of 95 °C for 1 min, 55 °C for 30 s, and 95 °C for 30 s. MiR-122, Gapdh, and Sesn2 sequences were obtained from the NCBI GenBank (Gene IDs: 100314023, 24383, and 502988, respectively). The primers (Sangon Biotech) used in this section were designed using the stem-loop method (miRNA) or Primer 5.0 software (gene) and are listed in Table 2. For data analysis, the comparative threshold cycle (CT) value of U6 (for miR-122) and Gapdh (for Sesn2) was used to normalize the loading variations in real-time PCR. The CT value of Sesn2/miR-122 minus that of Gapdh/U6 was determined as the ΔCT value. The ΔΔCT values were then obtained by subtracting the control ΔCT values from the corresponding experimental ΔCT values and converted into a fold difference using the equation 2^−ΔΔCT^.

**Table 2:** Primers used in quantitative qRT-PCR

Primers	Sequence 5′ → 3′
miR-122 RT^a^ primer	GTCGTATCCAGTGCAGGGTCCGAGGTGCACTGGATACGACCAAACAC
miR-122 forward primer	TGCGGTTGGAGTGTGACAATGG
miR-122 reverse primer	CAGTGCAGGGTCCGAGGT
U6 RT^a^ primer	CGCTTCACGAATTTGCGTGTCAT
U6 forward primer	GCTTCGGCAGCACATATACTAAAAT
U6 reverse primer	CGCTTCACGAATTTGCGTGTCAT
*Gapdh* forward primer	CATCACCATCTTCCAGGAGCG
*Gapdh* reverse primer	TGACCTTGCCCACAGCCTTG
*Sesn2* forward primer	AACCTTTCGTGCCCAGGATT
*Sesn2* reverse primer	GGAGCATGGAGGTGTCTACG

a RT, reverse transcription.

### Western blot

Total protein was extracted and lysed with cell lysis buffer (Bestbio, Cat# BB-3201-2). Protein concentration was then measured using the BCA Protein Quantitation Assay. Next, equal amounts of protein from different groups were separated by sodium dodecyl sulfate-polyacrylamide gel electrophoresis (SDS-PAGE) and transferred to a polyvinylidene difluoride (PVDF) membrane. The membrane was then blocked with TBST buffer for 2 h and incubated with primary antibodies against SESN2 and BETA-ACTIN at 4 °C overnight. After washing with TBST buffer for 3 times, the membrane was incubated with IgG HRP-conjugated secondary antibody for 2 h. Chemiluminescence (ECL) was used for image developing.

### Dual-luciferase reporter assay

The pmiR-RB-REPORT™ Vector (RiboBio) inserted with the wild-type (pmiR-RB-REPORT-Sesn2-wt) or mutant Sesn2 3′ UTR (pmiR-RB-REPORT-Sesn2-mut) or the vector itself (pmiR-RB-REPORT-Sesn2-si) were co-transfected into H9c2 cells with miR-122 mimics or NC in 24-well plates. Twenty-four hours after transfection, the renilla and firefly luciferase activities were measured using the Dual-luciferase reporter assay system according to the manufacturer's instructions. Firefly luciferase activity was used as the internal control and the normalized data were calculated as the quotient of renilla/firefly luciferase activities.

### Rat apoptosis RT^2^ profiler PCR array

First, the RNA samples were extracted using the RNeasy® mini kit and converted into first-strand cDNA using the RT^2^ first strand kit. Next, the cDNA was mixed with the RT^2^ SYBR Green Mastermix and aliquoted into the wells of the RT2 profiler PCR array. The PCR program was as follows: 1 cycle of 95 °C for 10 min; 40 cycles of 95 °C for 15 s, and 60 °C for 1 min; and 1 cycle of 95 °C for 1 min, 55 °C for 30 s, and 95 °C for 30 s. The relative mRNA level of the genes were compared between the treatment group and control group and presented as table and scatter plot. The total RT^2^ profiler PCR array gene expression analysis report was obtained using QIAGEN analysis (https://www.qiagen.com/cn/shop/genes-and-pathways/data-analysis-center-overview-page/) and provided in ESI.†

### Statistical analyses

All the experiments were performed at least 3 times. The data were analyzed using SPSS 19.0 and presented as mean ± standard deviation (SD). Differences between multiple groups were analyzed by one way or two way ANOVA followed by Dunnett post-tests (compared all groups with control group) or Bonferroni post-tests (compared all pairs of groups). Difference between two groups were analyzed by Student's two tailed *t* test. *P* < 0.05 was considered statistically significant.

## Results

### Cardioprotective effect of ghrelin on Ang II-induced H9c2 cell apoptosis

**Fig. 1:**
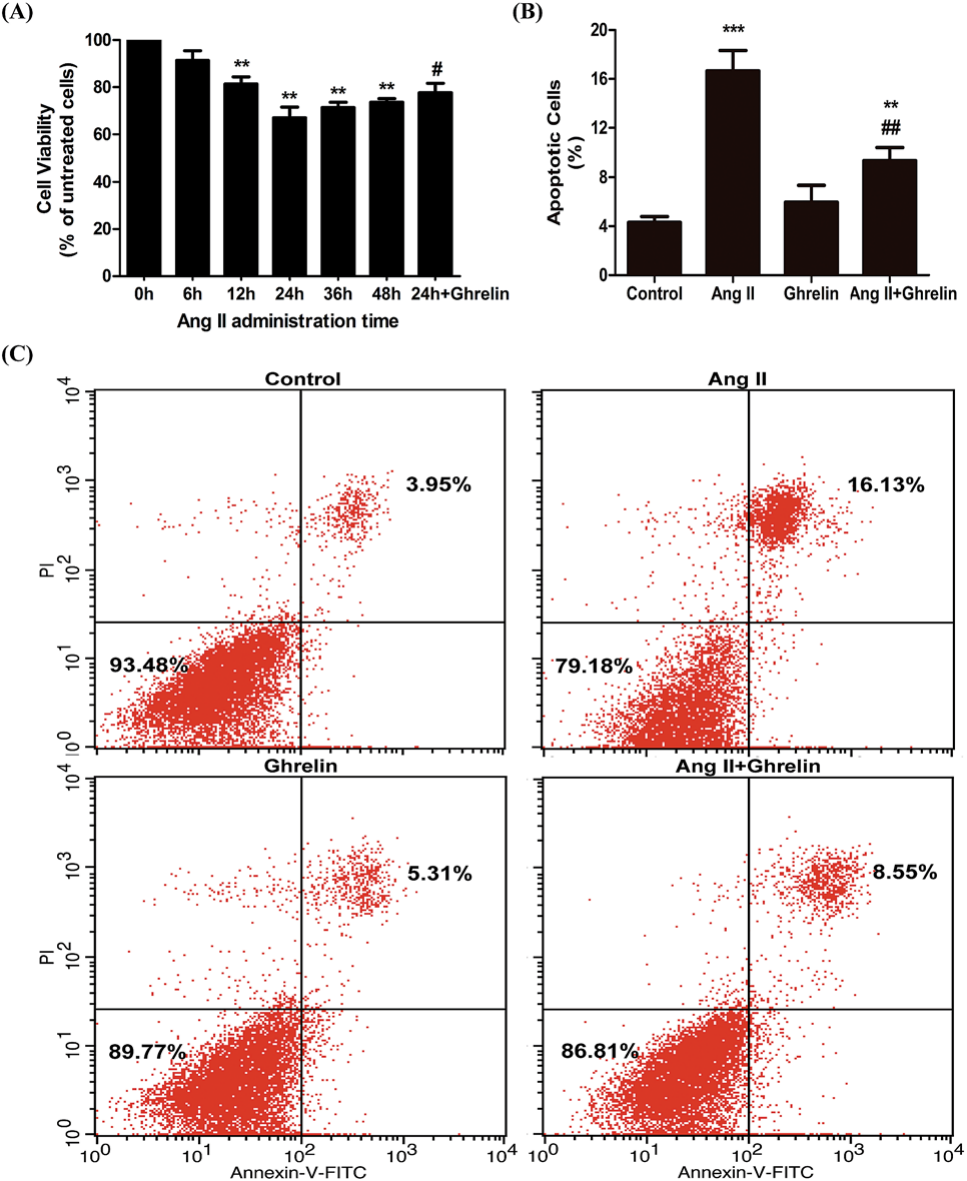
Ang II-induced H9c2 cells apoptosis and the cardioprotective role of ghrelin. (A) H9c2 treated with or without 100 nM ghrelin were incubated with 100 nM Ang II from 0 h to 48 h. Cell viability was examined by MTT assay and defined as the absorbance of the treated cells *versus* that of the untreated cells. (B), (C) H9c2 cells incubated in culture medium (control), ghrelin (100 nM), Ang II (100 nM) or both for 24 hours were stained with Annexin V-FITC/propidium iodide (PI) and analyzed by flow cytometry. **P < 0.01, ***P < 0.001 *versus* 0 h group or control group; ^#^*P* < 0.05, ^##^*P* < 0.01 *versus* Ang II 24 h group (*n* = 3).

To examine the effect of ghrelin on Ang II-induced apoptosis, H9c2 cells treated with or without 100 nM ghrelin were incubated with 100 nM Ang II for different durations. Cell viability was measured using the MTT assay. Compared with 0 h group, the groups subjected to Ang II treatment for 6–24 h showed decreased cell viability (range: 91.3 ± 4.2% to 67 ± 4.6%); cell viability began to recover after 24 hours of treatment. Consequently, we chose 24 h as the best duration for Ang II treatment; under this condition, ghrelin (100 nM) treatment increased cell viability to 77.7 ± 4% (Fig. 1(A)). To further investigate the effect of Ang II and ghrelin on cell apoptosis, the exact percentage of apoptotic cells was confirmed by Annexin V-FITC/propidium iodide (PI) double staining, followed by flow cytometry analysis. The results shown in Fig. 1(B) and (C) reveal that 24 h of Ang II treatment remarkably induced apoptosis (16.7 ± 1.7%; compared to control group: 4.3 ± 0.5%), and this deleterious effect could be significantly ameliorated by ghrelin treatment (percentage of apoptotic cells reduced to 9.4 ± 1%).

### Effects of Ang II and ghrelin on miR-122 expression

**Fig. 2:**
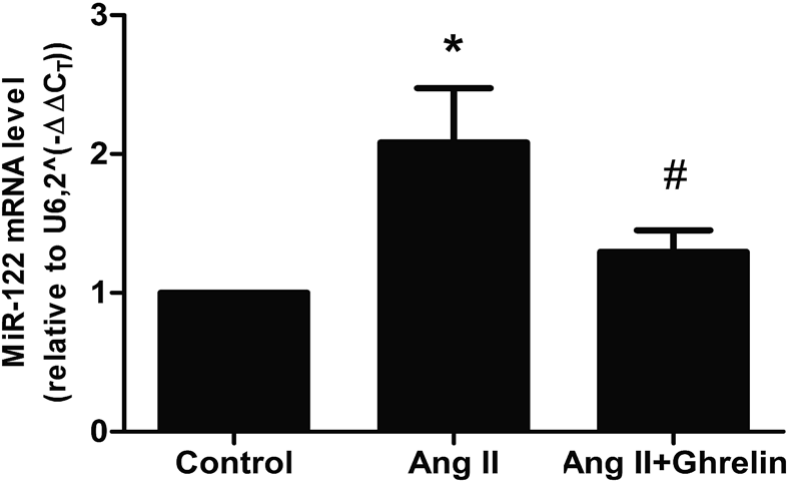
MiR-122 mRNA level was affected by AngII and ghrelin. H9c2 cells were incubated with culture medium (control), Ang II (100 nM), ghrelin (100 nM) or Ang II + ghrelin (100 nM) for 24 h. Total RNA was extracted and reversed using primers in Table 2. Relative mRNA level of miR-122 was detected by quantitative RT-PCR and normalized to U6. *P < 0.05 *versus* control group; ^#^*P* < 0.05 *versus* Ang II group (*n* = 3).

To investigate whether Ang II and ghrelin affected miR-122 expression, the relative mRNA level of miR-122 was detected by real-time RT-PCR, and the results normalized to U6 were calculated as 2^(−ΔΔCT)^. The primers used are shown in Table 2. miR-122 expression was significantly upregulated upon Ang II treatment (2.1 ± 0.4), and ghrelin treatment ameliorated the Ang II-induced upregulation of miR-122 (Fig. 2).

### Treatment with miR-122 inhibitors attenuated Ang II-induced apoptosis and enhanced the cardioprotective effect of ghrelin

**Fig. 3:**
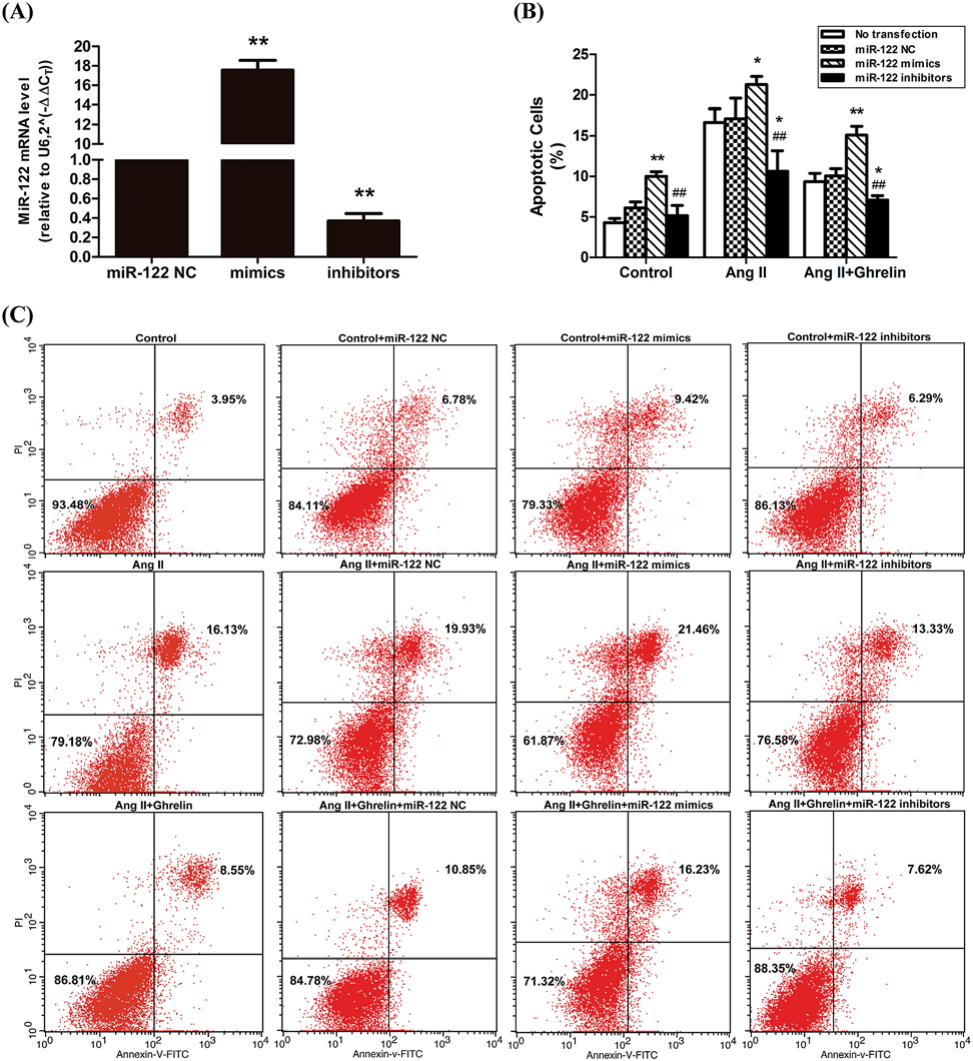
The impact of miR-122 on Ang II-induced apoptosis and cardioprotective effect of ghrelin. (A) MiR-122 mimics, inhibitors and NC (synthesized by Gene Pharma) were transfected into H9c2 cells using Fugene transfection reagent, the efficiency was examined by qRT-PCR; (B), (C) the successfully trasfected cells were incubated with culture medium, Ang II or Ang II + ghrelin for 24 h. The percentage of apoptotic cells was detected by flow cytometry. *P < 0.05, **P < 0.01 *versus* NC group; ^#^*P* < 0.05, ^##^*P* < 0.01 *versus* mimics group (*n* = 3).

H9c2 cells were transfected with miR-122 negative control (NC), miR-122 mimics, and miR-122 inhibitors. The transfection efficiency was verified by qRT-PCR: cells transfected successfully would have the associated change in miR-122 mRNA level (mimics increase mRNA level and inhibitors decrease mRNA level; Fig. 3(A)). The successfully transfected cells were then incubated in culture medium (control), 100 nM Ang II, and 100 nM Ang II + ghrelin for 24 h. The percentage of apoptotic cells was detected by flow cytometry. Cells transfected with miR-122 NC showed no significant difference compared to those without transfection, revealing that the transfection reagents and process hardly affects the H9c2 cells. Compared with the NC groups, all the mimics groups showed significantly increased cell apoptosis. The inhibitors group all showed significant differences compared with the mimics groups. Furthermore, miR-122 inhibitors significantly suppressed the apoptosis of H9c2 cells exposed to Ang II with (10.6 ± 2.5%) or without ghrelin (7.1 ± 0.6%) (compared with NC: 17.1 ± 2.5% and 10.1 ± 0.9%, respectively; Fig. 3(B) and (C)).

### PCR array analysis of the effect of miR-122 on apoptosis pathway

**Fig. 4:**
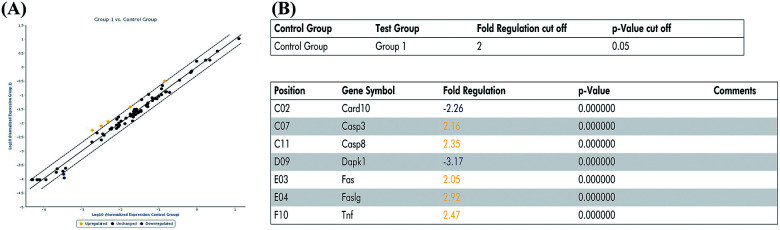
MiR-122 affected various apoptosis pathway. (A) H9c2 cells were transfected with miR-122 mimics or NC vector, extracted by RNeasy® mini kit, and reversed by RT^2^ first strand kit followed by real-time fluorescent quantitative PCR. The normalized expressions of every gene on the array between miR-122 mimics (group 1) and NC group (control group) were compared and presented as scatter plot. The central line indicated unchanged gene expression. The dotted lines indicate the selected fold regulation threshold. Data points beyond the dotted lines in the upper left and lower right sections meet the selected fold regulation threshold. (B) The differentially expressed genes are listed with name and fold regulation. Fold-regulation represents fold-change results in a biologically meaningful way. Fold-change (2^(−Delta Delta CT)^) is the normalized gene expression (2^(−Delta CT)^) in the test sample divided the normalized gene expression (2^(−Delta CT)^) in the control sample (see ESI† data sheet for more details).

In the previous experiment, we found that miR-122 mimics significantly increased apoptosis, while treatment with miR-122 inhibitors protected the H9c2 cells from apoptosis. Next, to identify the signaling pathway involved in miR-122-mediated apoptosis, cells transfected with miR-122 mimics and NC were extracted and analyzed by QIAGEN apoptosis RT^2^ profiler PCR array. According to the QIAGEN analysis software, miR-122 over-expression upregulated the expression of death-receptor-mediated pathway-related genes including Fas, Faslg, and Tnf, and also induced the expression of caspase cell apoptosis pathway-related genes including Casp3 and Casp8, while downregulating Dapk1 and Card10, which show anti-apoptotic effects (Fig. 4). These data further revealed miR-122 plays a key role in the regulation of cell apoptosis.

### Sesn2 is a direct target of miR-122

**Fig. 5:**
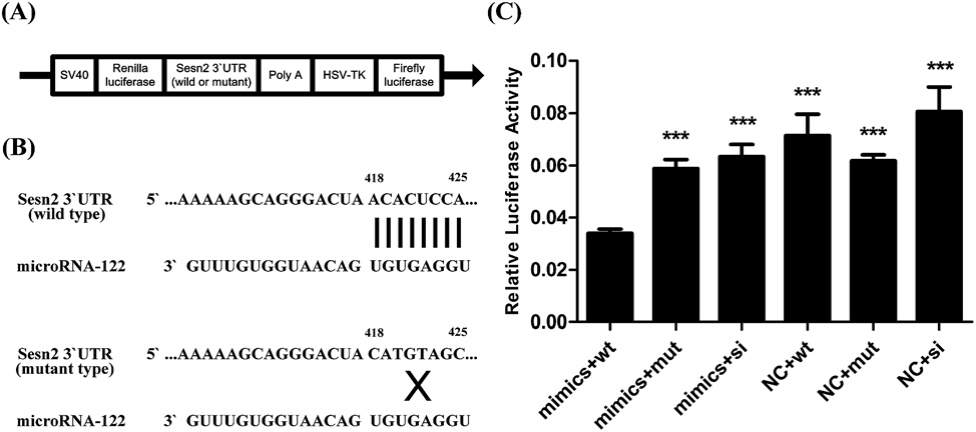
Sesn2 is a direct target of miR-122. Cells transfected with pmiR-RB-REPORT-Sesn2-wt, pmiR-RB-REPORT-Sesn2-mut or pmiR-RB-REPORT-Sesn2-si vector would simultaneously be transfected by miR-122 mimics or miR-122 NC. (A) Construct maps of the vectors mentioned above. (B) Schematic diagram of miR-122 binding sites in wild or mutant type of Sesn2 3′ UTR. (C) The renilla luciferase activity was measured by the Dual-Luciferase Reporter Assay System and normalized to firefly luciferase activity. ***P < 0.001 *versus* pmiR-RB-REPORT-Sesn2-wt + miR-122 mimics group (*n* = 3).

Sesn2 was predicted to be the target gene of miR-122 using TargetScan human7.1; the schematic diagram of miR-122 binding sites in the Sesn2 3′ UTR is given in Fig. 5(B). To verify whether miR-122 regulates Sesn2 directly through binding to its 3′ UTR, we cloned wild-type (pmiR-RB-REPORT-Sesn2-wt) and mutant (pmiR-RB-REPORT-Sesn2-mut) Sesn2 3′ UTR into the pmiR-RB-REPORT™ vector (RiboBio, Guangzhou, China) (Fig. 5(A)). The dual-luciferase assay was then applied to investigate the role of miR-122 in regulating Sesn2. If a microRNA pairs to the sequence cloned to the downstream of the renilla luciferase gene in the vector, the renilla luciferase activity will be attenuated, but firefly luciferase activity will not change. In our study, relative luciferase activity was significantly inhibited upon co-transfection with pmiR-RB-REPORT-Sesn2-wt and miR-122 mimics group (0.034 ± 0.002) compared with that in the other groups (Fig. 5(C)), which clearly revealed that Sesn2 is a direct target of miR-122.

### MiR-122 negatively regulated Sesn2 in H9c2 cells

**Fig. 6:**
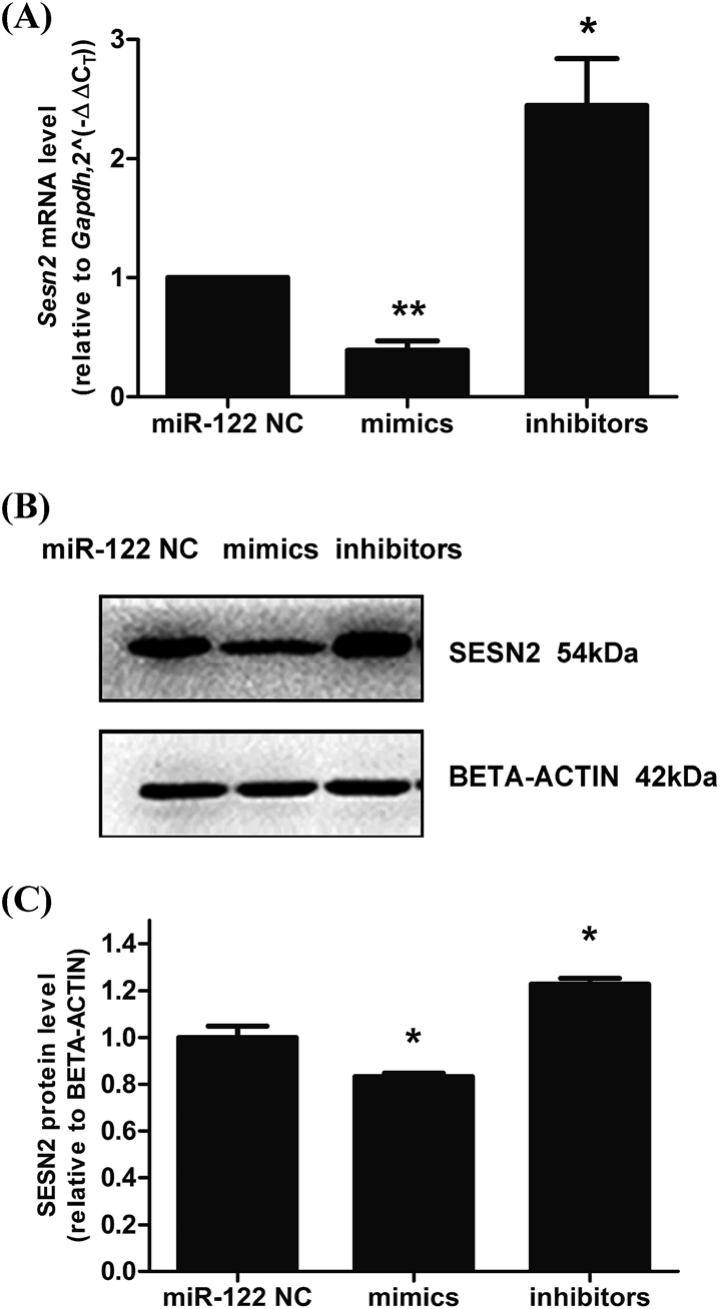
MiR-122 negatively regulated Sesn2 expression in H9c2 cells. MiR-122 mimics, inhibitors and NC were transfected into H9c2 cells. The total RNA and protein were extracted after 24 h transfection. (A) The mRNA level of Sesn2 was detected by qRT-PCR; (B) a representation of Western Blot gel showing SESN2 protein expression was downregulated in mimics group and upregulated in inhibitors group; (C) an analysis data of Western blotting. *P < 0.05, **P < 0.01 *versus* NC group (*n* = 3).

Next, to verify the correlation between miR-122 and Sesn2 in cells, H9c2s were transfected with miR-122 NC, miR-122 mimics, and miR-122 inhibitors, and the mRNA and protein levels of Sesn2 were detected by real-time RT-PCR and western blot, respectively. As shown in Fig. 6, both the mRNA and protein levels of Sesn2 increased in the miR-122 inhibitors group (2.44 ± 0.4 and 1.23 ± 0.03, respectively) and decreased in the miR-122 mimics group (0.39 ± 0.08 and 0.83 ± 0.02, respectively) compared with that in the NC group, thus suggesting that miR-122 negatively regulates Sesn2 mRNA and protein expression.

### Effects of Ang II and ghrelin on Sesn2 expression

**Fig. 7:**
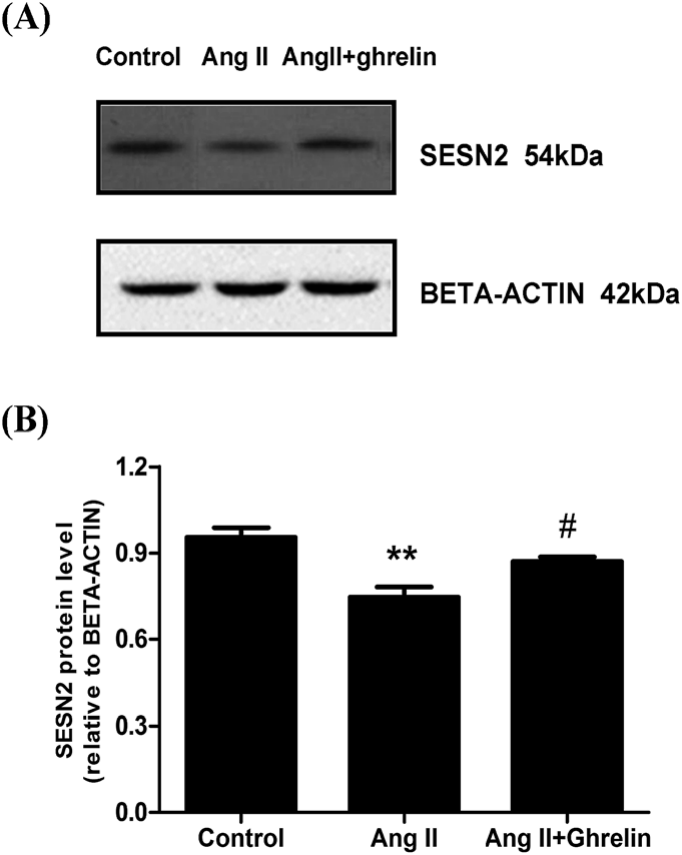
The effect of Ang II and ghrelin on Sesn2 expression. H9c2 cells were incubated with culture medium (control), Ang II (100 nM) or Ang II + ghrelin (100 nM) for 24 h. (A) A representation of Western Blot gel showing SESN2 protein expression was suppressed by Ang II administration and rescued by ghrelin co-treatment; (B) an analysis data of Western Blot. **P < 0.01 *versus* control group; ^#^*P* < 0.05 *versus* Ang II group (*n* = 3).

Since miR-122 expression could be affected by Ang II and ghrelin, we further investigated the effect of Ang II and ghrelin stimulation on Sesn2 expression. Western blot showed that Ang II significantly decreased Sesn2 protein level when compared with control group while co-treatment with ghrelin increased Sesn2 protein level when compared with Ang II group (Fig. 7(A) and (B)).

### Exogenous expression of Sesn2 alleviated Ang II insult and facilitated ghrelin protection

**Fig. 8:**
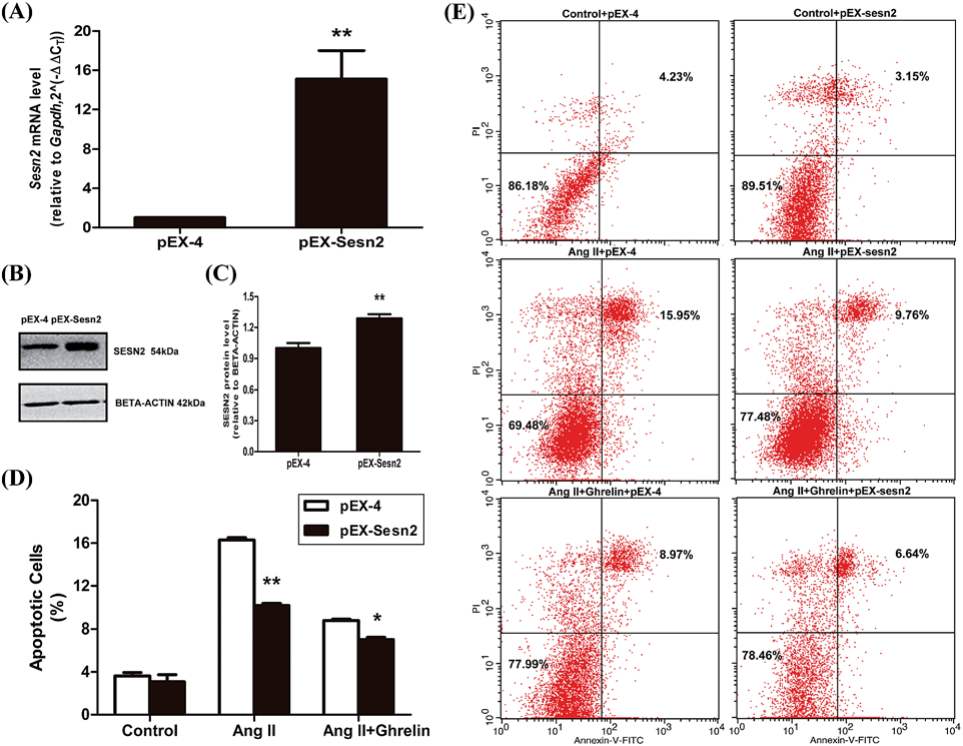
Overexpression of Sesn2 alleviated Ang II-induced cell apoptosis and enhanced the protective role of ghrelin. (A) The qRT-PCR data of cells transfected with overexpression vector (pEX-4) and the vector inserted with Sesn2 cDNA (pEX-Sesn2); (B) a representation of Western Blot gel showing SESN2 protein expression was upregulated by pEX-Sesn2; (C) an analysis data of Western Blot; (D), (E) H9c2 cells transfected with pEX-4 and pEX-sesn2 were incubated with culture medium (control), Ang II (100 nM) or Ang II + ghrelin (100 nM). The percentage of apoptotic cells was detected by flow cytometry. *P < 0.05, **P < 0.01 *versus* pEX-4 group (*n* = 3).

Overexpression of miR-122 increased Ang II-induced apoptosis and attenuated the cardioprotective effect of ghrelin. We predicted that the target gene of miR-122, Sesn2, might play a role in this effect of miR-122. Therefore, we constructed a Sesn2 overexpression vector (pEX-Sesn2) and transfected it into H9c2 cells. Transfection efficiency was confirmed by qRT-PCR and western blot (Fig. 8(A)–(C)). The effect of Sesn2 gain-of-function on H9c2 apoptosis was assessed by flow cytometry. Exogenous overexpression of Sesn2 significantly reduced Ang II-induced cell apoptosis (10.2 ± 0.4% *versus* 16.3 ± 0.3% in the vector control pEX-4 group), and this decrease was accompanied by an enhanced protective effect of ghrelin (7.0 ± 0.3% *versus* 8.7 ± 0.2% in the vector control pEX-4 group). However, Sesn2 overexpression only slightly changed the percentage of apoptotic cells without any additional treatment (no Ang II or ghrelin) (Fig. 8(D) and (E)).

## Discussion

There are considerable studies focused on the importance of gradual cardiomyocyte loss in the development of heart failure. Myocardial apoptosis is one of the most important modes of cardiomyocyte loss^36^ and has been associated with the activation of the RAS system. Ang II, a key modulator of the RAS, is known to regulate apoptosis, hypertrophy, myocardial contraction, and cardiac remodeling.^5^ Ghrelin, a novel peptide hormone, has been shown to exert cardioprotective effects including improving endothelial function, reducing vascular tension, and increasing cardiac output; however, the most important mode is inhibiting cardiomyocyte apoptosis. Ghrelin was first discovered as the endogenous ligand for GHS-R^6^ and the canonical pathway through which acylated ghrelin exert its cardioprotective effects is binding to GHSR-1a. The first four residues are minimal necessary sequence and the *n*-octanoic acid modification of serine 3 is required for the activation of GHSR-1a.^37^ In this study, we found that acylated ghrelin protected H9c2 cells from Ang II-induced apoptosis, which was consistent with our previous studies.^38,39^ We further attempted to determine the underlying mechanism. Previously, Zhang and Shu^11^ reported that acylated ghrelin could upregulate miR-21 to ameliorate ischemia/reperfusion-induced injury, which led us to hypothesize that ghrelin with the essential *n*-octanoylated serine 3 residue targets a miRNA to inhibit Ang II-induced apoptosis.

MiR-122 acts as a biomarker for acute coronary syndrome (ACS) and acute myocardial infarction (AMI) diagnosis.^25–28^ A previous study has shown that miR-122 regulates apoptosis in cutaneous T-Cell lymphoma through the p53/Akt signaling pathway.^40^ Additionally, miR-122 has been found to promote cardiac apoptosis in Pax-8-/- mice and to affect CCK-8 expression in H9c2 cells.^21^ In our previous study, we used high-throughput sequencing to develop a differential miRNA profile in a rat model of post-infarction heart failure and found that miR-122 expression was highly increased in the pathological model.^24^ A similar increase was also obtained upon exposure to Ang II, and reversed upon ghrelin treatment, which revealed that miR-122 could be regulated by Ang II and ghrelin and may involve in Ang II-induced apoptosis and ghrelin protection. To investigate this, we conducted flow cytometry analysis and concluded that miR-122 mimics significantly increased cell apoptosis while miR-122 inhibitors reversed this fatal effect in Ang II and Ang II + ghrelin group when compared with miR-122 NC. This indicated that miR-122 plays a vital role in cardioprotective effect of ghrelin on Ang II-induced cell apoptosis and inhibition of miR-122 can facilitate ghrelin protection. Furthermore, we investigated the potential apoptotic signaling pathways affected by miR-122 using PCR array, a novel and reliable tool for analyzing the expression of a focused panel of genes and for studying cell signaling pathways.^41^ We found that miR-122 upregulated the expression of Casp3, Casp8, Fas, Faslg, and Tnf, which are mainly involved in caspase cascade reaction and death receptor-mediated pathways such as the Fas and Tnf signaling pathways, which are critical in cell apoptosis. However, further studies are needed such as detecting key molecules of these pathways to confirm which pathway plays a dominant role.

We also predicted Sesn2 which prevents cardiomyocyte apoptosis as the target gene of miR-122 using TargetScan human7.1 (http://www.targetscan.org/vert_71/). Both the mRNA and protein levels of Sesn2 were dramatically downregulated in the miR-122 mimics group and upregulated in the inhibitors group. Moreover, the relative luciferase activity significantly decreased in the H9c2 cells cotransfected with pmiR-RB-REPORT-wt vector (containing Sesn2 3′ UTR) and miR-122 mimics, which clearly demonstrated the target relationship between miR-122 and Sesn2. Furthermore, pEX-4 and pEX-Sesn2 were transfected into H9c2 cells, followed by treatment with Ang II and ghrelin. Significantly lower apoptosis was observed in both the Ang II group and the Ang II + ghrelin group compared with that in the pEX-4 group. Combined with the result that Sesn2 protein level was remarkably downregulated by Ang II and upregulated by ghrelin, we supposed that Sesn2 inhibition by miR-122 was involved in Ang II-induced apoptosis and in the cardioprotective effect of ghrelin. We speculate that a similar reduction of apoptosis was not observed in the control group, mainly because Sesn2 is a stress-inducible gene. Therefore, when no external treatment is given to the cells, no significant change will be observed even when Sesn2 is overexpressed.

In recent years, it was reported that unacylated ghrelin, another form of ghrelin, could also regulate miR-221/222 expression to reduce ischemia-induced tissue damage in a mouse model of peripheral artery disease.^42^ Whether unacylated ghrelin protects cells from Ang II insult *via* miR-122/Sesn2 pathway and the indispensability of the *n*-octanoylated serine 3 residue in this process still need more investigation. In summary, our study showed that Sesn2 is the direct target of miR-122 and their expression is under the regulation of Ang II and ghrelin. Furthermore, we found that miR-122 inhibitors and Sesn2 overexpression alleviate Ang II-induced H9c2 cell apoptosis and enhance the protective effect of ghrelin. We also showed that miR-122 influences several apoptosis pathways including the death receptor-mediated pathway and caspase cascade reaction pathway.

## Conclusions

Collectively, our data revealed miR-122 and its target gene Sesn2 are significant players in cardiomyocyte apoptosis and can be therefore be developed as therapeutic targets. Further *in vivo* studies such as endogenous inhibition of miR-122 and overexpression of Sesn2 are needed.

## Conflicts of interest

There are no conflicts to declare.

## Supplementary Material
